# SARS-CoV-2 infection mediates differential expression of human endogenous retroviruses and long interspersed nuclear elements

**DOI:** 10.1172/jci.insight.147170

**Published:** 2021-12-22

**Authors:** Jez L. Marston, Matthew Greenig, Manvendra Singh, Matthew L. Bendall, Rodrigo R.R. Duarte, Cédric Feschotte, Luis P. Iñiguez, Douglas F. Nixon

**Affiliations:** 1 Division of Infectious Diseases, Weill Cornell Medicine, New York, New York, USA.; 2 Department of Molecular Biology & Genetics, Cornell University, Ithaca, New York, USA.

**Keywords:** COVID-19, Infectious disease, Innate immunity

## Abstract

SARS-CoV-2 promotes an imbalanced host response that underlies the development and severity of COVID-19. Infections with viruses are known to modulate transposable elements (TEs), which can exert downstream effects by modulating host gene expression, innate immune sensing, or activities encoded by their protein products. We investigated the impact of SARS-CoV-2 infection on TE expression using RNA-Seq data from cell lines and from primary patient samples. Using a bioinformatics tool, Telescope, we showed that SARS-CoV-2 infection led to upregulation or downregulation of TE transcripts, a subset of which differed from cells infected with SARS, Middle East respiratory syndrome coronavirus (MERS-CoV or MERS), influenza A virus (IAV), respiratory syncytial virus (RSV), and human parainfluenza virus type 3 (HPIV3). Differential expression of key retroelements specifically identified distinct virus families, such as Coronaviridae, with unique retroelement expression subdividing viral species. Analysis of ChIP-Seq data showed that TEs differentially expressed in SARS-CoV-2 infection were enriched for binding sites for transcription factors involved in immune responses and for pioneer transcription factors. In samples from patients with COVID-19, there was significant TE overexpression in bronchoalveolar lavage fluid and downregulation in PBMCs. Thus, although the host gene transcriptome is altered by infection with SARS-CoV-2, the retrotranscriptome may contain the most distinctive features of the cellular response to SARS-CoV-2 infection.

## Graphical abstract

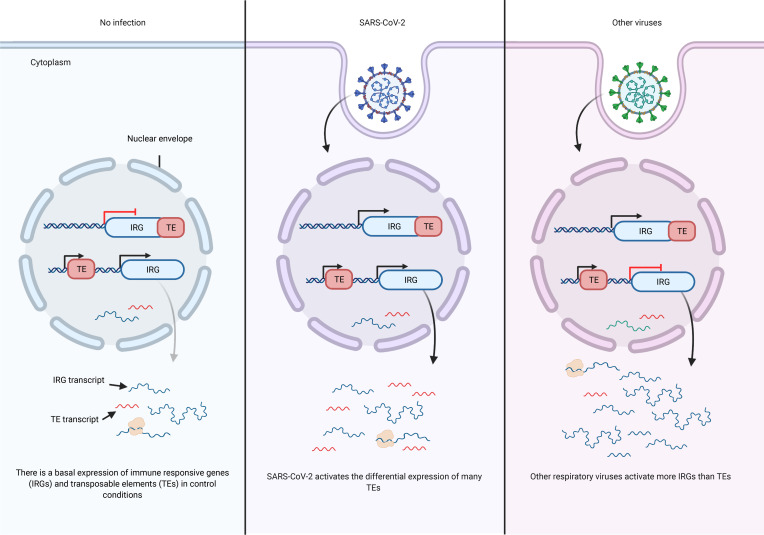

## Introduction

SARS-CoV-2 infection has caused a worldwide pandemic with many millions of people infected. The development and distribution of SARS-CoV-2–specific vaccines have helped to suppress new infections, but a rising number of variant strains threaten global efforts to achieve sufficient protective immunity. Since the identification of SARS-CoV-2, the search for new or repurposed drugs that treat COVID-19 has been disappointing, with a limited number of drugs currently authorized for emergency use. A better understanding of viral immunopathogenesis could identify new avenues for treatments. Current studies have identified viral replication at the beginning of infection and a latter exuberant host immune response as 2 major opportunities for clinical intervention (1).

Upon infection with a virus, cell host and viral genes act separately and in tandem to promote viral replication as well as initiate a host cell response. Coronaviruses, including SARS-CoV-2, hijack host cellular machinery and gene expression networks in order to enhance their own replication within the cell (2, 3). During this process, viral genetic material and proteins serve as signals to elicit innate and adaptive immune responses (4–7). Although a recent study has recognized the co-option of a transposable element (TE) sequence for differential isoform usage in the expression of the ACE2 receptor in the response to SARS-CoV-2 infection (8), most coronavirus studies to date have ignored the potential contribution of TEs in disease pathogenesis.

Human endogenous retroviruses (HERVs) and long interspersed nuclear elements type 1 (LINE-1) are TEs that contribute numerous repeat sequences throughout the human genome. Despite comprising approximately 25% of the genome, these repetitive sequences are often disregarded in genomic and transcriptional studies. However, the noncoding and coding activities of these retroelements impinge on a variety of cellular processes that can directly influence physiological responses, including innate sensing, and disease states (9). EBV (10) and HIV (11) are 2 viruses known to modulate TEs with downstream effects on immunopathogenesis, but how coronaviruses, particularly SARS-CoV-2, change TE expression to influence disease pathology and clinical course is a new area of research (12–14).

Previous research has demonstrated significant variation in the expression of HERVs in response to different viral infections in human and mouse models (15). LINE-1 elements have also been implicated in the pathogenesis and inhibition of viral infections (16). Therefore, we hypothesized that differentially expressed TEs in SARS-CoV-2 infection could reveal new mechanisms underlying the pathogenesis of COVID-19 and direct further research into new therapeutic opportunities. Here, we analyzed publicly available RNA-Seq data of cell lines infected with the SARS-CoV-2, SARS-CoV or SARS, Middle East respiratory syndrome coronavirus (MERS-CoV or MERS), influenza A virus (IAV), respiratory syncytial virus (RSV), and human parainfluenza virus type 3 (HPIV3) to determine virus-specific signatures in the retrotranscriptome. We further analyzed RNA-Seq data obtained from clinical samples of patients infected with SARS-CoV-2. We found that differential expression of key retroelements could identify a virus family, such as Coronaviridae, with unique retroelement expression subdividing viral species. Thus, the retrotranscriptome may contain the most distinctive features of host response to SARS-CoV-2 infection and provide new insights into pathogenesis and treatment of COVID-19.

## Results

### Differential expression of TEs after SARS-CoV-2 infection.

In order to elucidate the effects of SARS-CoV-2 infection on the human retrotranscriptome, we quantified the expression of TEs from publicly available RNA-Seq data of SARS-CoV-2–infected pulmonary cell lines (17, 18) and primary COVID-19 patient-derived samples (19) using the specialized bioinformatics tool Telescope (20) (Table 1 and Supplemental Table 1; supplemental material available online with this article; https://doi.org/10.1172/jci.insight.147170DS1). Telescope applies an expectation-maximization algorithm to reassign multiple mapped reads to a specific location, allowing a genomic locus-specific resolution of TE expression. We assessed differential expression of retroelements, as well as genes, and compared retrotranscriptomic changes from SARS-CoV-2 infection with those induced by related pathogenic human coronaviruses, SARS and MERS, and by other common respiratory viruses, IAV, RSV, and HPIV3. As described in Blanco-Melo et al. (17), A549 cells were found to be relatively nonpermissive to SARS-CoV-2 infection in contrast to Calu-3 cells given their lower expression of the SARS-CoV-2 host receptor ACE2. We therefore also compared the retrotranscriptomes of A549 cells transduced with a vector expressing human ACE2 receptor (A549-ACE2) at different SARS-CoV-2 MOIs in order to determine the effects of viral burden on the expression of retroelements. We also compared the retrotranscriptomes of A549 cells infected at the higher MOI with those pretreated with the kinase inhibitor ruxolitinib to determine the effects of IFN-I signaling and JAK/STAT pathway interference on retroelement expression. Blanco-Melo et al. (2020) showed that inhibition of this pathway reduced the expression of IFN-stimulated genes (ISGs) and enhanced viral replication; however, this intervention did not inhibit the release of inflammatory cytokines (17).

We first performed a principal component analysis (PCA) on the transcriptomic and retrotranscriptomic data from Calu-3, A549, and A549-ACE2 cells and found that the infected samples clustered separately depending on the type of virus infection, even between closely related coronaviruses (Supplemental Figure 1). This result demonstrated that infected cells not only underwent virus-specific changes in gene expression but also in their expression of HERVs and LINE-1 retroelements.

**Figure 1:**
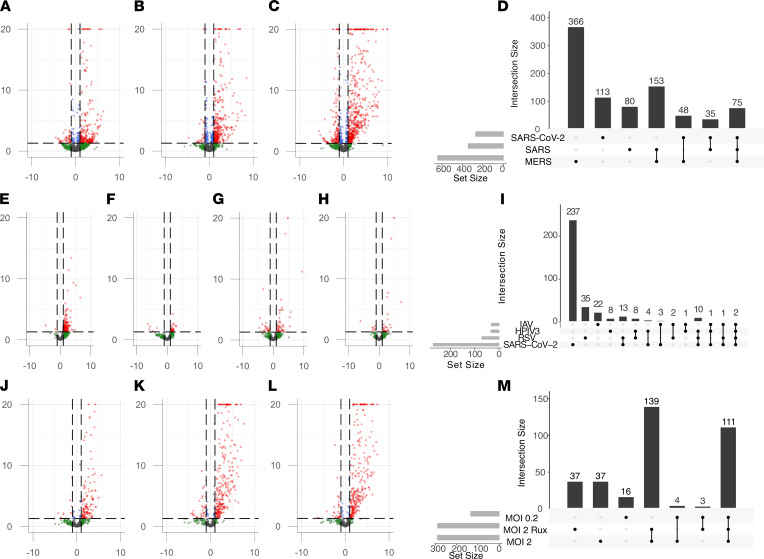
Differential retrotranscriptome profiling of in vitro viral infection of lung adenocarcinoma cell lines (Calu-3, A549, and A549-ACE2). Volcano plots of differential retroelement expression induced by related human coronaviruses in Calu-3 cells (**A**–**D**), various respiratory virus infections in A549 cells (**E**–**I**), and SARS-CoV-2 infection of A549-ACE2 cells at different MOIs and pretreated with ruxolitinib kinase inhibitor (**J**–**M**).

In Calu-3 cells, the 3 coronavirus infections (SARS-CoV-2, SARS, and MERS) each elicited unique profiles of retroelement expression (Figure 1, A–D, and Supplemental Table 2). For SARS-CoV-2 infection, there were 92 upregulated retroelements (52 HERVs and 40 LINE-1 elements), of which 14 HERVs and 7 LINE-1 elements were identified to be intergenic. Since many retroelements intersect human genes, the upregulation of intergenic retroelements indicates the presence of transcripts uniquely derived from retroelement sequences in response to infection. Of note, ERV316A3_6p22.3c, an HERV that was upregulated only with SARS-CoV-2 infection, intersects *HCP5*, a long noncoding RNA (lncRNA) previously reported to be driven by the HERV promoter (21). Additionally, a set of 72 retroelements were upregulated across all 3 coronavirus infections, including 40 HERVs and 32 LINE-1 elements, and of these, 13 HERVs and 4 LINE-1 elements were intergenic (Figure 1D).

We performed a TE family enrichment of all elements differentially expressed after each coronavirus infection. The results showed a shared enrichment of retroelements from the HERVE and HARLEQUIN families and SARS-CoV-2 specifically enriched for differential expression of HERVH family and HERV3 family loci (adjusted *P* < 0.05) (Supplemental Figure 2, A and B). Comparing the differential expression of these enriched HERV families showed that each virus induced unique changes in expression at these loci, including those that were upregulated (HERVH_4q31.3c) or downregulated (HERVH_11q24.1c) across all 3 infections, as well as those specific for SARS-CoV-2, including HERVH_15q26.3b, HARLEQUIN_7q33a, HERV3_7q33, HERVH_13q33.3, and HERVH_4p15.33b, among others (Supplemental Figure 2B).

In A549 cells, SARS-CoV-2, RSV, HPIV3, and IAV all induced differential expression of individual TEs. As with Calu-3 cells, each viral infection in A549 cells was associated with its own virus-specific retrotranscriptomic profile. Interestingly, the greatest number of differentially expressed TE loci occurred with SARS-CoV-2 infection, followed by RSV and then HPIV3, despite low permissiveness to infection. Intriguingly, IAV infection only induced limited changes in TE expression (Figure 1, E–I, and Supplemental Table 3). Two elements had differential expression for all 4 viral infections — MER4_22q12.3 and L1FLnI_7q21.2o — which intersect with the human genes *APOL6* and *SAMD9*, respectively. MER4_22q12.3 contributes to the 3′-UTR of *APOL6* and therefore may provide a polyA signal to the end of this ISG. The L1FLnI_7q21.2o sequence is antisense to *SAMD9* but contributes a large portion of the gene’s intron and second exon. Both genes are IFN responsive, which may explain the expression of intersecting retroelements; however, the contributions of these retroelement sequences to these ISGs remains to be studied (22, 23). Ten retroelements were differentially expressed in SARS-CoV-2, RSV, or HPIV3 infections, but not with IAV (Figure 1I). These included 3 elements that belong to the ERV316A3 family located on chromosomes 2, 6, and 12, including the *HCP5*-intersecting locus. The response to SARS-CoV-2 infection included the overexpression of 91 elements, of which 51 were HERVs and 40 were LINE-1 elements.

In A549-ACE2 cells infected with SARS-CoV-2, there was a substantial increase in differentially expressed TEs in comparison to nontransduced A549 cells (Figure 1, J–M, and Supplemental Table 4). Of note, a larger number of TE loci were upregulated than downregulated, as observed in other cell lines. A comparison of A549-ACE2 cells infected with SARS-CoV-2 at 2 different MOIs showed that more than 100 TEs were differentially expressed at either MOI, but there was a greater differential expression at the higher MOI. One hundred forty-five retroelements were upregulated at an MOI of 2 not seen at the lower MOI of 0.2, including 17 intergenic HERVs and 8 intergenic LINE-1 elements. *HCP5* was shown to be upregulated at the higher and not the lower MOI, suggesting a threshold of viral burden to transactivate its expression. Administration of the JAK/STAT pathway inhibitor ruxolitinib prior to SARS-CoV-2 infection also altered the TE expression response, but the majority of differentially expressed retroelements were shared across the different MOI and treatment conditions. However, ruxolitinib treatment inhibited the upregulated expression of 21 retroelements, including 6 HERVs and 15 LINE-1 elements. Of the 6 HERVs, 4 were intergenic sequences with no overlapping human coding genes and an annotated HERV-derived gene *ERV3-1*, overlapping with the *ZNF117* gene and HERV4_4q22.1. The changes in the expression response of these intragenic HERV transcripts suggests independent transcriptional regulation of these loci by JAK/STAT signaling pathways.

Although each model system showed distinct profiles, there were shared elements in all 3 in vitro models of untreated SARS-CoV-2 infection (Calu-3, A549, and A549-ACE2 at both MOIs) with upregulation of 18 common retroelements, including 9 HERVs and 9 LINE-1 elements, of which 3 HERVs were intergenic, including HARLEQUIN_7q33a, HERV3_7q33, and HML6_6p22.2, suggesting locus-specific transactivation of these HERVs upon SARS-CoV-2 infection. Of note, both ERV316A3_6p21.33c (*HCP5*) and MER4_22q12.3 (3′-UTR of *APOL6*) were upregulated across all 3 cell models of SARS-CoV-2 infection (Supplemental Figure 3 and Supplemental Table 5).

### Associations between the SARS-CoV-2–induced retrotranscriptome and innate immune genes.

Given the significant dysregulation of key cytokines, such as IL-6 and TNF-α, in patients with COVID-19, we analyzed the RNA-Seq data from these in vitro infection models and compared the data with samples from patients with COVID-19 for similar immune gene expression signatures. SARS-CoV-2 significantly upregulated the expression of downstream NF-κB–regulated innate immune genes, such as *IL6*, *IL1B*, and *TNF* in Calu-3 cells (24). However, upregulation of these *IL1B* transcripts was not seen in SARS or MERS infection of Calu3 cells (Supplemental Figure 4). We also compared 3 host immune genes with intersecting HERV loci differentially expressed in SARS-CoV-2 infection, *APOL6*, *HCP5*, and *SP140L* (Supplemental Figure 4). We showed that these intersected genes were also differentially upregulated in SARS-CoV-2 infection alongside the identified retroelement loci, MER4_22q12.3, ERV316A3_6p21.33c, and ERV316A3_2q37.1a, respectively.

We then explored associations between the SARS-CoV-2–induced retrotranscriptome and key innate immune gene expression changes in host cells. We applied the GREAT analysis tool, which assigns biological meaning to a set of noncoding or TE regions by analyzing nearby gene annotations, to the differentially expressed TE sequences induced by SARS-CoV-2, SARS, and MERS infection in Calu-3 cells (25). From this analysis, Gene Ontology (GO) terms were derived from enriched host genes. There was a marked enrichment for GO terms pertaining to type I IFN signaling pathway, defense response to virus, innate immune response, and IFN-γ–mediated signaling pathway terms (Supplemental Table 5). The differentially expressed genomic regions were associated with numerous immune genes, including *OAS2*, *OAS3*, *IFNAR1*, *IL10RB*, *SP100*, *HLA-B*, *HLA-G*, *HLA-V*, *FCGR2A*, *IL6*, *APOL5*, *APOL6*, *JAK2*, *MX1*, and *MX2* (Supplemental Table 6). Despite this enrichment in SARS-CoV-2 infection, no GO terms were enriched from differentially expressed TEs from SARS or MERS infections. The set of differentially expressed retroelement loci from the other cell types did not show enrichment for specific GO sets through the GREAT analysis. This observation may be due to differences in permissibility to SARS-CoV-2 infection as noted in Blanco-Melo et al. (17).

### Immune response genes and specific retroelements are upregulated in SARS-CoV-2 infection.

**Figure 2:**
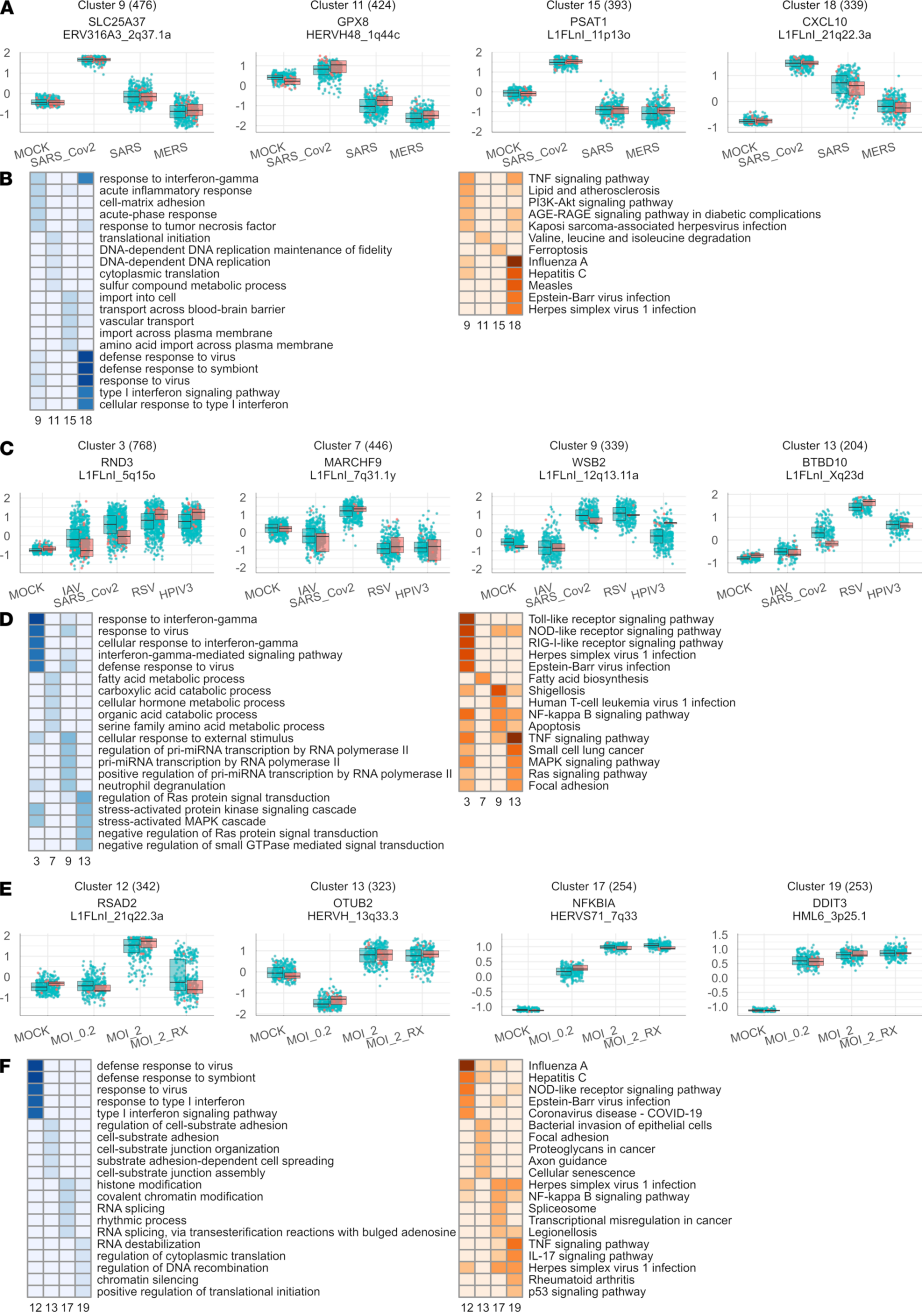
Network analysis of gene and retroelement expression changes induced by various viral infections. (**A**) Selected gene clusters produced from the comparison of the transcriptomes of Calu-3 cells in 4 infection conditions (MOCK; SARS_Cov2; SARS; MERS). For each cluster, the scaled mean expression value (*z* score) for each gene in each group is plotted as a point. Box plots show the median expression *z* score (dark line) and the first and third quartiles (edges) of the cluster’s gene expression values in each condition. (**B**) Overrepresentation analysis of Kyoto Encyclopedia of Genes and Genomes (KEGG) pathways (left) and Gene Ontology (GO) terms (right) for selected Calu-3 clusters. Terms in the heatmap are shaded according to –log10(*P*), using the *P* value obtained from a hypergeometric test for each term’s overrepresentation in each cluster’s set of genes. Terms with *P* > 0.05 are not shaded. (**C**) Selected gene clusters from the comparison of the transcriptomes of A549 cells in 5 infection conditions (MOCK; SARS_Cov2; IAV; RSV; HPIV3). (**D**) KEGG and GO enrichment analysis results for selected A549 cell clusters. (**E**) Selected gene clusters from the comparison of the transcriptomes of A549-ACE2 cells in 4 infection conditions (MOCK; MOI 0.2; MOI 2; MOI 2 + ruxolitinib). (**F**) KEGG and GO enrichment analysis results for selected A549-ACE2 cell clusters.

To explore the specific gene and retroelement networks that underlie the host response to SARS-CoV-2 infection, we applied hierarchical clustering to identify sets of genes (clusters) with significantly correlated expression patterns among the infections of Calu-3, A549, and A549-ACE2 cells. We filtered all genes based on statistically significant differential expression patterns identified by the likelihood ratio test for infection (adjusted *P* < 0.05), which produced 31 clusters of genes and coexpressed retroelements in Calu-3 cells, 13 clusters in A549 cells, and 18 clusters in A549-ACE2 cells (Figure 2 and Supplemental Figures 5–7).

Importantly, each clustering analysis highlighted a cluster of genes and retroelements that were induced by SARS-CoV-2 infection and enriched for GO terms, including defense response to virus, response to interferon gamma, and cellular response to type I interferon. Additionally, these clusters also enriched for Kyoto Encyclopedia of Genes and Genomes (KEGG) pathways induced by virus infection, including IAV, HCV, EBV, HSV-1, and measles (Figure 2, A and B). From the Calu-3 cluster 18, 4 intergenic HERVs were correlated with antiviral gene expression, ERV316A3_12q24.13 (between OAS2 and OAS3 genes), ERV316A3_4p15.2c, HERVIP10F_14q11.2c, and HML1_10q22.1. In A549 cells, 2 intergenic HERVs correlated with the antiviral gene cluster expression, HERV3_11q13.3, annotated as LINC02701, and HERVL_9p24.1b (Figure 2, C and D). No intergenic HERVs were associated with the antiviral response in A549-ACE2 cells (Figure 2, E and F). However, in A549-ACE2 cells, treatment with ruxolitinib decreased expression of retroelements that intersect with human genes involved in the response to virus cluster 12. This suggested coordinated expression of these retroelements in the signaling pathways involved in this immune response (Supplemental Table 7).

### Transcription factor interactions with differentially expressed TE loci.

**Figure 3:**
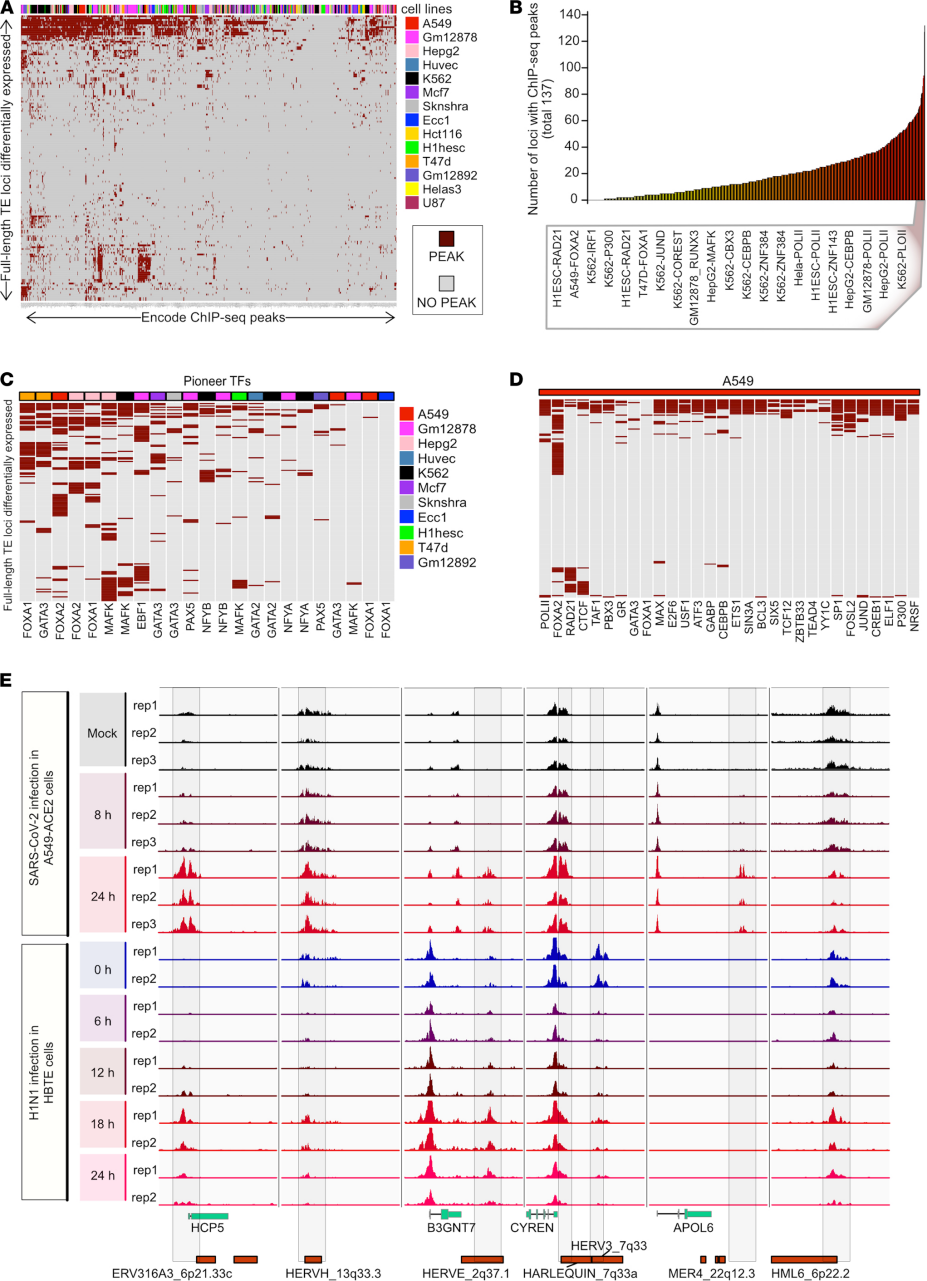
ChIP-Seq analysis of SARS-CoV-2–induced differentially expressed TEs. (**A**) Heatmap summarizing the transcription factor (TF) occupancy over individual differentially expressed (DE) TE proviral loci in 14 human cell lines. This plot includes the distinct DE-TE loci that are occupied by at least 1 ChIP-Seq peak. Each row represents an individual DE-TE locus. Each column shows TF ChIP-Seq peak occupancy of a different cell line. Gray color denotes the absence, whereas dark red color denotes the presence of peaks in each locus. TFs are annotated on the top of the heatmap and shown in a legend on the right side. (**B**) Bar plot shows the number of DE-TE loci occupied by each ChIP-Seq in each cell line analyzed in **A**. The bars are shown in ascending order according to the number of enriched DE-TE loci. The ChIP-Seq shown in the box occupy at least half of the total DE-TE loci. (**C**) Heatmap shows the subsection of analysis in **A** while only including the attested and predicted pioneer TFs. The color scheme of the plot and legends is the same as in **A**. (**D**) This heatmap shows the subsection of analysis in **A**, including only the transcriptional- and enhancer-associated factors in A549 cells. The color scheme of the plot is the same as in **A**. (**E**) Genome browser tracks show the distribution of ChIP-Seq signal of H3K27Ac around the DE-TE loci (7 kb upstream until 3 kb downstream sequences from the putative transcription start site) in A549 cells after SARS-CoV-2 infection at the time points 0, 8, and 24 hours (upper panel) and H3K27Ac signals at the same set of loci shown in human bronchial tracheal epithelium cells after the H1N1 infection at time points, namely 6, 12, 18, and 24 hours (lower panel).

To further investigate the independent expression of TEs in response to viral infection, we first analyzed differentially expressed TEs seen in SARS-CoV-2 infection of Calu-3 cells for binding of transcription factors based on ChIP-Seq data produced as part of the ENCODE project (26). We observed an enrichment of polymerase II peaks within the TE sequences themselves, suggesting that the TEs were expressed independently of overlapping host genes. Transcription factors identified in the response to SARS-CoV-2 infection, such as JUND, FOSL2, and FOXA2, and chromatin double strand break and remodeling factors, such as CTCF, YY1, and RAD21, were enriched for binding to the TE loci identified from our Telescope analysis (Figure 3, A–D). We focused on binding enrichment for pioneer transcription factors that bind closed chromatin and induce chromatin opening, such as FOXA and GATA3 (27, 28). Of the differentially expressed retroelements, the top 20 retroelements with enrichment for pioneer factor binding sites in A549 cells included HERV3_7q33, HERVH_13q33.3, and HML6_6p22.2, which were significantly upregulated across SARS-CoV-2 infections (Supplemental Table 8).

We next used ChIP-Seq data for the histone acetylation mark H3K27Ac, which correlates with transcriptional activity (29), to profile the regions surrounding the differentially expressed TE loci during SARS-CoV-2 infection of A549 cells (30). We observed an increased accumulation of this histone mark over time in the region immediately upstream of the differentially expressed TEs, suggesting chromatin remodeling of these TEs to an active state after 24 hours of SARS-CoV-2 infection (Figure 3E). This pattern was observed for a diverse set of TEs and genomic contexts, including an ERV316A3 element that contributes to *HCP5*; an HERVE element downstream of the *B3GNT7* gene; a MER4 element within the 3′-UTR of *APOL6*; and 4 intergenic HERV sequences, HERVH_13q33.3, HARLEQUIN_7q33a, HERV3_7q33, and HML6_6p22.2 (Supplemental Figure 8, A–D).

In addition, we analyzed the compendium of publicly available data of 1000 ChIP-Seq–derived transcription factor binding sites (TFBSs) provided by ReMap2020 (31). By comparing the annotated TFBSs within the genomic regions of the SARS-CoV-2–induced retroelements against the full set of TFBSs with all retroelement regions in the genome, we noted an enrichment of binding sites for transcription factors involved in immune responses (RELA, IRF5, and BCL3), chromatin structural regulation (MPHOSPH8, CHAMP1, and TRIM28), and transcriptional activation (JUND and TBP). This comparison further suggests that these retroelements were subject to chromatin remodeling upon infection (Supplemental Figure 9).

In order to investigate the viral specificity of this epigenetic response, we then compared H3K27ac data over the time course of H1N1 influenza virus infection of human bronchial tracheal epithelium cells, a primary cell model for pulmonary viral infection (32). Similar to our findings for SARS-CoV-2 infection in A549 cells, we found an increase of this histone mark over time in the region upstream of the same set of differentially expressed TEs (Figure 3E). Last, the HERVE_2q37.1 integrant downstream of the *B3GNT7* gene was also acetylated after influenza infection and harbored the regulatory sequence of its neighboring human gene. A subset of differentially expressed TE loci underwent similar epigenetic changes after influenza and SARS-CoV-2 infection, possibly leading to their overexpression.

### SARS-CoV-2–induced retrotranscriptome changes in primary clinical samples.

**Figure 4:**
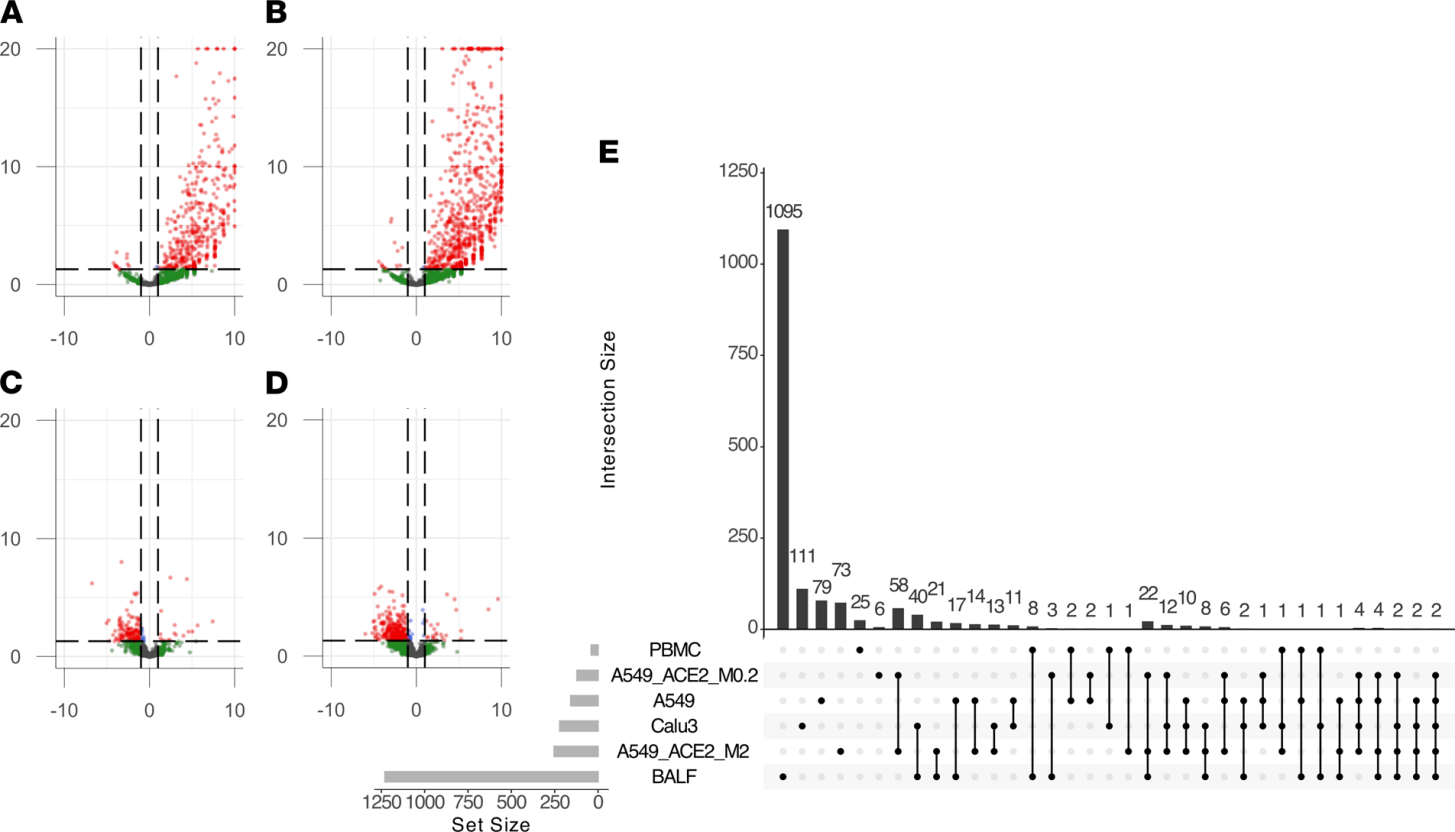
Differential retrotranscriptome profiling of RNA-Seq data from clinical samples from donors with COVID-19. Volcano plots of differentially expressed TEs from BALF samples from donors with COVID-19 compared with healthy donors separated by (**A**) HERVs and (**B**) LINE-1 elements. Volcano plots of differentially expressed TEs from PBMCs of patients with COVID-19 compared with healthy donors separated by (**C**) HERVs and (**D**) LINE-1 elements. (**E**) Upset plot of differentially expressed retroelement intersections across SARS-CoV-2 (COV2) infections of Calu-3 cells, A549 cells, A549-ACE2 cells, and BALF and PBMCs from clinical samples of patients with severe COVID-19.

In order to explore whether these findings were replicated in clinical samples, we analyzed publicly available bulk RNA-Seq data of bronchoalveolar lavage fluid (BALF) and PBMCs from patients hospitalized with severe COVID-19 (19) compared with samples from healthy donor controls. The results showed a profound dysregulation in the expression of retroelements (Figure 4, A–D), with significant overexpression in BALF and downregulation in PBMCs. We conducted a PCA based upon these retrotranscriptomic profiles and found that the infected samples clustered separately from the healthy clinical samples, with the infected BALF samples showing more profound changes than those of PBMCs (Supplemental Figure 10 and Supplemental Table 9). Our comparison of the lung tissue–derived samples (Calu-3, A549, A549-ACE2, and BALF) found 1 LINE-1 transcript differentially expressed across all SARS-CoV-2 infections, L1FLnI_16p11.2i (Figure 4E); however, this observed change in expression may be due to differential expression of the ZNF267 gene within which this LINE-1 element is contained.

Given recent findings of differential expression of a pathogenic HERVW family member in the leukocytes of patients with COVID-19 (12), we sought to determine whether the PBMCs show differential expression of HERVW transcripts. In PBMCs, we saw a marked upregulation of HERVW_15q21.2, which intersects the *GLDN* gene. However, no open reading frames encoding an envelope protein were determined from this locus. Interestingly, BALF, which may also contain numerous immune cells, showed a marked upregulation of HERVW_7q21.2, also known as *ERVW-1*, which encodes an envelope protein, syncytin-1, known to mediate the formation of syncytia, specifically in placental syncytiotrophoblasts (33, 34). Another HERW locus, HERVW_4q21.22, which lies intergenic between *ENOPH1* and *TMEM150C*, was upregulated in BALF and can produce an annotated transcript, XM_011532442.2, which is annotated as a “protein-coding,” “syncytin-1–like” transcript, LOC105377310. The transcript contains an open reading frame for a 245-residue envelope protein with 85.37% sequence homology to syncytin-1 by BLAST search. Similarly, HERVFRD_6p24.2, which encodes syncytin-2, an envelope protein from the HERVFRD family also involved in placental syncytiotrophoblast fusion (35), was upregulated in the BALF samples of patients with COVID-19. Thus, our analysis suggests that several HERV protein products may have been overexpressed in BALF of patients with COVID-19.

## Discussion

In this study, we describe the impact of SARS-CoV-2 infection on the transcription of locus-specific endogenous retrotransposons, specifically HERV and LINE-1 elements. This level of retrotranscriptomic precision provides insights into the potential genomic effects of exogenous viral and host immune factors on the activation of endogenous retrotransposon sequences. Given the extensive literature on the co-option of endogenous retroviruses as immune-responsive *cis*-regulatory elements (36–38), this study provides a quantitative analysis of retrotransposon expression patterns from publicly available RNA-Seq data in response to viral infections, with a focus on the specific TE responses to SARS-CoV-2 infection.

We quantified and analyzed the retrotranscriptome of SARS-CoV-2 infection in in vitro cell lines, in comparison to SARS and MERS and to other common respiratory viruses IAV, RSV, and HPIV3. SARS-CoV-2 induced the expression of a unique retrotranscriptomic signature in comparison with related coronavirus infections in Calu-3 cells and in comparison with other common respiratory virus infections in A549 cells. The retrotranscriptome of SARS-CoV-2 differed between each cell type, suggesting that retrotranscriptomic activation in response to virus is both virus specific and cell type specific. Furthermore, there was marked upregulation of TEs in BALF but not from PBMCs from patients with COVID-19.

In SARS-CoV-2–infected A549-ACE2 cells, a higher MOI induced upregulation and downregulation of a larger number of retroelements, suggesting that the induction of certain elements may be dependent on the level of viral burden. Pretreatment of A549-ACE2 cells with ruxolitinib also showed many similarities to untreated cells, suggesting that the retrotranscriptomic response to SARS-CoV-2 in the A549-ACE2 cells was largely reproducible and consistent and that the expression of a small number of retroelements was altered by pretreatment with the kinase inhibitor. However, many of these elements intersected known IFN and immune response genes, which may account for the changes in expression of these retroelements. The finding that ruxolitinib effectively reduced the expression of ISGs in vitro is significant in the context of SARS-CoV-2 infection, and this pharmacological intervention is being tested in the RUXCOVID clinical trial, which did not meet the primary endpoint of reducing the number of hospitalized patients with COVID-19 who experienced severe complications (39). For this study, although the expression of retroelements in response to SARS-CoV-2 infection was largely unchanged by pretreatment with ruxolitinib, there is an interesting similarity in unchanged cytokine expression found in the Blanco-Melo et al. study (17). This finding may suggest that SARS-CoV-2–induced retroelements correspond more with inflammatory cytokine expression dynamics than with the IFN-stimulated response. Perhaps the expression of these elements may themselves be cytokine-like mediators of inflammation (40).

In Calu-3 cells, we showed that SARS-CoV-2 induced an elevated expression of HERVs from the HERVH and HERV3 families (not enriched in SARS or MERS infections) and shared enrichment of HARLEQUIN and HERVE families across virus types. This result suggests a common induction of TE families after coronavirus infections and specifically for SARS-CoV-2. Additionally, using the GREAT analysis tool, we showed that the set of retroelements that was differentially expressed in response to SARS-CoV-2 infection of Calu-3 cells was enriched in genomic regions proximal to human genes involved in the innate immune response, response to virus, and response to IFN-γ pathways. This finding suggests that these elements may play roles in the expression or modulation of these immune genes (17, 36) or may themselves be transcribed alongside these immune response genes. This genomic enrichment was unique to the differentially expressed retroelements induced by SARS-CoV-2 in Calu-3 cells and not in other viral infections or in the A549 or A549-ACE2 infections with SARS-CoV-2, which may be due to the differences in A549 permissibility to SARS-CoV-2 infection (17).

A set of upregulated retroelements was shared across the cell types infected with SARS-CoV-2, which implies that these elements may play a role in the infection cycle or host response to SARS-CoV-2 infection. Many of these elements were also intergenic elements and may be activated independently from host genes by SARS-CoV-2 infection. Of particular note, the HARLEQUIN_7q33a and HERV3_7q33 transcripts, which may be expressed as individual or mosaic transcripts, were upregulated across all infections. The same HERV3 locus has previously been described as a marker of response to immunotherapy in various cancers (41) and lies downstream of the *CYREN* gene, of which an alternatively spliced transcript encodes the modulator of retrovirus infection (MRI-1) micropeptide (42). Additionally, an intergenic HERVK family member transcript, HML6_6p22.2, was also upregulated across the SARS-CoV-2–infected cells. The HERVK family has been implicated in various disease pathologies (43–45). Further investigation will seek to characterize the expression, regulation, and coding potential of these elements to determine their contributions to physiology and disease. Finally, this analysis points a spotlight on 2 human genes, *HCP5* and *APOL6*, whose transcripts contain HERV-derived sequences and are upregulated in SARS-CoV-2 infection. Both genes have been shown to be involved in the immune response, but further characterization is warranted in connection with SARS-CoV-2 infection and the contribution of their intrinsic HERV sequence components (46).

By applying a likelihood ratio test to determine differentially expressed genes for downstream coexpression clustering and analysis, we showed that differentially expressed retroelements coclustered with many gene networks in response to the various viral infections in each cell type tested. Notably, we identified the set of retroelement transcripts that were coexpressed with clusters that were enriched for genes involved in the defense response to virus, response to type I IFN, and response to IFN-γ GO terms and KEGG pathways related to various viral infections. This adds support to the hypothesis that certain retroelements may be coactivated with or play roles in the immune response to virus.

One common caveat of the current analysis — and of retrotranscriptomic analysis in general (47) — is that many retroelement sequences are nearby or overlap with previously described protein-coding or noncoding genes. Thus, expression of TE loci may not be mechanistically related to retroelement sequences but in fact derive from transcriptional regulation of nearby genes. However, we posit that observed expression of TE loci can be and often is a property of the TE locus and should be considered together with analysis of other genes. First, we have shown that repetitive TE loci are a rich source of *cis*-regulatory elements (4). Second, TE sequences themselves encode functional elements, including peptides and RNAs. For example, HCP5 is an antisense retroviral transcript that has been co-opted by the innate immune system through an HLA class I promoter (48). Many of the differentially expressed retroelements were also intergenic, suggesting that changes in transcription were independent of any intersected human genes.

We also addressed whether these retroelements were being independently transcribed in response to viral infection by analyzing the transcriptional and epigenetic regulation of these genomic sequences in response to virus infection. From an analysis of TFBS enrichment for the set of retroelements differentially expressed in SARS-CoV-2 infection of Calu-3 cells, we showed that a portion of these elements had extensive ChIP-Seq peaks from transcription initiation factors, suggesting the potential for independent transcription. Additionally, this enrichment was seen for the same retroelements induced in available data from A549 cells. We then compared histone acetylation marks (H3K27Ac) to analyze chromatin alterations around the set of differentially expressed retroelements in publicly available ChIP-Seq data of SARS-CoV-2 infection of A549 cells and, to assess the potential for broad response to other viruses, H1N1 infection of primary human bronchial tracheal epithelium tissue. We showed that after various time points after infection, the histone acetylation changed around these differentially expressed retroelements in both SARS-CoV-2 infection of A549 cells and H1N1 infection of human bronchial tracheal epithelium, with a notable increase in the size of the H3K27Ac peaks 18–24 hours after infection. We also demonstrated that the genomic sequences of the differentially expressed retroelements in SARS-CoV-2 were enriched for the NF-κB subunits RELA and RELB, implicating this immune response pathway in modulating retroelement expression.

By analyzing the retrotranscriptomes of primary BALF and PBMC samples from hospitalized patients with COVID-19, we showed that profound changes in the retrotranscriptome correlate with disease. In the BALF samples, there was a marked upregulation of retroelement expression, similar to that seen in cell lines, and some of these elements are shared across the in vitro lung adenocarcinoma cell infections. In contrast to the BALF samples, there was a marked downregulation of retroelement expression in the PBMCs from patients with COVID-19. This result was striking and may be explained by differences in permissibility to viral infection of blood cells. In the case of low SARS-CoV-2 infection of blood cells, this differential expression may instead be induced by either the inflammatory or cytokine-enriched environment resulting from infection of other permissible tissues. This hypothesis should be tested with further studies of blood retrotranscriptomes in response to inflammation or isolated viral infection. Although the BALF samples shared some upregulated retroelements with the in vitro models, the PBMCs showed less similarity, suggesting tissue specificity in retroelement induction.

From our analysis of clinical samples of COVID-19, we also showed marked upregulation of HERV-derived envelope transcripts encoding syncytin-1 and syncytin-2, as well as another HERVW transcript with an open reading frame encoding an envelope protein in BALF. In light of findings of a HERVW envelope protein detected in leukocytes of patients with COVID-19 (12), the presence of syncytium in infected lung tissue (49), and the fusogenic properties of HERV-derived synctin-1 and syncytin-2 (33–35), we present data that might support the presence of HERVW envelope transcription in response to infection. These HERV transcripts may play roles in the development of syncytium and the pathophysiology of the inflammatory milieu in COVID-19. Although these proteins are immunogenic, many retroviral envelope proteins also have been noted to have immunosuppressive functions (50, 51). Although the immunosuppressive function of syncytin-1 is still debated, syncytin-2 has been demonstrated to dampen cytokine production (52). Therefore, the presence of these retroviral transcripts and their protein products may also contribute to the delayed IFN response noted in SARS-CoV-2 infection and the inflammatory dynamics of COVID-19 (17). Only 1 HERVW transcript was shown to be upregulated in PBMCs from patients with COVID-19 when compared with healthy donors; however, the transcript did not contain an open reading frame for an envelope protein. This finding may be affected by the heterogeneity of the PBMC samples and the lower permissibility to SARS-CoV-2 in peripheral blood cells. A major limitation of these findings, however, is that the BALF samples from healthy donors were also from a different study than the samples derived from patients with COVID-19 and may be affected by batch effects.

In summary, differential expression of TEs after viral infections could provide insights into disease pathogenesis and unique viral signatures in viral diagnostics. As the biology of locus-specific TEs develops, the interplay of TEs, genes, and viral effects will add significant knowledge to the biology of viral infections.

## Methods

### Samples.

In order to compare the expression of host gene and retroelement transcripts from different viral infection models, publicly available data from in vitro infection models of SARS-CoV-2 infection in Calu-3 cells described by Blanco-Melo et al. (17) were retrieved from National Center for Biotechnology Information’s (NCBI) Sequence Read Archive (SRA) with the following accession numbers: SRR11517744, SRR11517745, SRR11517746, SRR11517747, SRR11517748, and SRR11517749. Infection models of A549 cells with SARS-CoV-2, IAV, RSV, and HPIV3 were retrieved with the following accession numbers: SRR11517674, SRR11517675, SRR11517676, SRR11517677, SRR11517678, SRR11517679, SRR11412279, SRR11412280, SRR11412287, SRR11412288, SRR11517750, SRR11517751, SRR11517752, SRR11517753, SRR11517754, SRR11517755, SRR11517756, SRR11517757, and SRR11517758. Data from different MOI SARS-CoV-2–infected A549-ACE2 cells and those pretreated and untreated with ruxolitinib at MOI of 2 were accessed with the following accession numbers: SRR11573892, SRR11573893, SRR11573894, SRR11573895, SRR11573896, SRR11573897, SRR11573898, SRR11573899, SRR11573900, SRR11573901, SRR11573902, SRR11573903, SRR11573904, SRR11573905, SRR11573906, SRR11573907, SRR11573908, SRR11573909, SRR11573910, SRR11573911, SRR11573912, SRR11573913, SRR11573914, SRR11573915, SRR11573916, SRR11573917, SRR11573918, SRR11573919, SRR11573920, SRR11573921, SRR11573922, SRR11573923, SRR11573924, SRR11573925, SRR11573926, and SRR11573927. Samples were run in quadruplicate, so raw FASTQ files for each sample were concatenated before alignment. RNA-Seq data of Calu-3 infection models of SARS and MERS as described in Yeung et al. (18) were retrieved from NCBI’s SRA with accession numbers SRR1942929, SRR1942934, SRR1942956, SRR1942957, SRR1942960, SRR1942961, SRR1942989, and SRR1942990. Finally, RNA-Seq data from clinical BALF samples of 2 patients with COVID-19 and PBMCs of 3 patients with COVID-19 were downloaded from the National Genomics Data Center-Genome Sequence Archive with the following accession numbers: CRR119890, CRR119891, CRR119892, CRR119893, CRR119894, CRR119895, CRR119896, CRR119897, CRR125445, and CRR125446. RNA-Seq data from BALF samples of healthy individuals (53) were downloaded from the NCBI’s SRA with accession numbers SRR10571724, SRR10571730, and SRR10571732, as compared in Xiong et al. (19).

### Transcriptome and retrotranscriptome quantification.

RNA-Seq data sets were downloaded and mapped to GENCODE V.33 transcriptome with Salmon for gene quantification. The retrotranscriptome expression was determined using Telescope. Briefly, reads were mapped with Bowtie2, allowing up to 100 multimapping positions and adjusting the parameters for local alignments (-very-sensitive-local, -k 100 –score-min L,0,1.6). Then, the mapping results were used as input for Telescope using a publicly available annotation of TEs, including HERV and LINE-1 sequences (https://github.com/mlbendall/telescope_annotation_db/tree/master/builds/retro.hg38.v1), for read reassignment. High-confidence transcript reads assigned to TE sequences in the annotation were used for further analysis.

### Filtering genes for clustering.

To identify genes exhibiting significant expression variation between experimental conditions, the likelihood ratio test from DESeq2 version 1.24.0 was applied separately to the set of retroelement and human transcript counts. For the transcriptomic data derived from Calu-3 cells, the likelihood of a full model containing coefficients for the 4 different infection conditions was compared with a reduced model containing only an intercept term for each gene. For the A549 cell data, a full model containing coefficients for the 5 conditions and sequencing batch was compared with a reduced model containing only the sequencing batch coefficient. Finally, this analysis was performed on A549-ACE2 cell data. In all analyses, genes with adjusted *P* values less than 0.05 were selected for downstream analyses. Human genes and retroelements that were deemed significant by this standard were combined into a single transcript count matrix for downstream analysis. Transcript counts for these genes were stabilized using DESeq2’s variance stabilizing transformation and scaled to zero mean and unit variance within each gene.

### Clustering.

The set of genes identified as statistically significant by the likelihood ratio test were clustered using average-linkage agglomerative hierarchical clustering with Pearson correlation distance (1 – Pearson’s correlation) as a dissimilarity metric. A dynamic tree cut (54) was subsequently applied to produce clusters of genes from the dendrogram, using a minimum cluster size of 200 genes for the A549 data and 300 genes for the Calu-3 data.

### Cluster analysis.

Cluster expression patterns were visualized using the R package ggplot2 (55), plotting each gene’s mean scaled expression value within each experimental condition. Cluster eigengenes were solved as the first principal component of each cluster’s scaled expression matrix. Cluster hub genes and hub retroelements were identified by calculating all pairwise Pearson correlations between genes in the cluster. The top *n* hub genes for each cluster were identified as the *n* human genes with the greatest mean correlation with all other genes — both human genes and retroelements — in the cluster. The hub retroelement in each cluster was identified as the retroelement with the greatest mean correlation with all other genes — both human genes and retroelements — in the cluster.

### Overrepresentation analysis.

Gene clusters were tested for overrepresentation of pathways from the KEGG and GO terms using the hypergeometric test implementation from the R package clusterProfiler (56) with *P* value cutoff of 0.05 and *q* value cutoff of 0.20. For each cluster selected for overrepresentation analysis, *P* values for the top 5 KEGG pathways and GO terms — ranked by adjusted *P* value —were visualized in a heatmap using the R package pheatmap. Terms and pathways that were also significantly overrepresented in other clusters (adjusted *P* < 0.05) were colored on the heatmap accordingly.

### GREAT analysis.

Differentially expressed retroelements from each of the infections and cell types were input as bed files into the GREAT analysis web tool (25). Only the set of differentially expressed retroelements from SARS-CoV-2 infection of Calu-3 cells showed proximity to genomic regions enriched for genes involved in GO terms associated with immune function.

### ReMap2020 transcription factor binding enrichment analysis.

The differentially expressed retroelements were then transformed to genomic range objects (GRanges) using the GRanges R package. Additionally, all retroelement loci in the Telescope annotation were transformed into GRanges objects. Using the ReMap2020 data set and R package, the differentially expressed retroelement loci were compared against the ReMap2020 annotated TFBSs within all Telescope-annotated retroelement loci for enrichment of specific transcription factors and plotted as enrichment dot plots with the ReMap2020 R package.

### ENCODE ChIP-Seq analysis.

ChIP-Seq data sets from ENCODE consortium, SARS-CoV-2 (GSE167528), and H1N1 infection (GSE113702) in SRA format were downloaded and converted to FASTQ format using sratools function fastq-dump -- split-3. FASTQ reads were mapped against the hg19 reference genome with the modified Bowtie2 parameters: -- very-sensitive-local. All unmapped reads, the mapped ones with MAPQ less than 10, and PCR duplicates were removed using Picard and samtools. MACS2 called all the ChIP-Seq peaks with the parameters -- nomodel -q 0.01 -B. Blocklisted regions were excluded from called peaks (https://www.encodeproject.org/annotations/ENCSR636HFF/). To generate a set of unique peaks, we merged the peaks using the mergeBed function from bedtools, where the distance between peaks was less than 50 bp. We then intersected these peak sets with the differentially expressed TEs from hg19 repeat-masked coordinates using bedtools intersectBed with a 50% overlap. To calculate the enrichment over the given repeat elements, we first extended 5 kb upstream and 5 kb downstream coordinates from the left boundary (transcription start site) of respective elements in a strand-specific manner. These 10 kb windows were further divided into 100 bp bins, and tags (bedGraph output from MACS2) were counted in each bin. Tag counts in each bin were normalized by the total number of tags per million in given samples and presented as cpm per 100 bp. We averaged cpm values for each bin between replicates before plotting the figures.

### Code availability.

All scripts, files, and data used for the analysis are publicly available on GitHub at https://github.com/LIniguez/Marston_etal_2021 (commit ID 38062e2) and https://github.com/mgreenig/HERV-cluster-analysis (commit ID 19b6e3c).

### Statistics.

Differential expression was calculated using Wald’s test for single comparisons, and genes were considered differentially expressed genes or TEs with an adjusted *P* value (FDR) < 0.05 and an absolute log_2_ fold change > 1. For clustering analysis, the likelihood ratio test was performed, and features were considered differentially expressed for further analysis if adjusted *P* value was less than 0.05 (FDR). For the enrichment analysis of TE families, we used a Fisher exact test to calculate *P* value, and families were considered enriched in the provided list if adjusted *P* value (FDR) was less than 0.05.

### Study approval.

The Weill Cornell Medicine IRB deemed this study not human subject research.

## Author contributions

JLM, MG, LPI, and DFN designed the study and wrote the manuscript. MG, MS, MLB, RRRD, and CF helped with study design, performed the data analysis, and wrote the manuscript. JLM, MG, MLB, LPI, and DFN conceived the research. All authors wrote and approved the manuscript.

## Supplementary Material

Supplemental data

Supplemental tables 1-9

## Figures and Tables

**Table 1:**
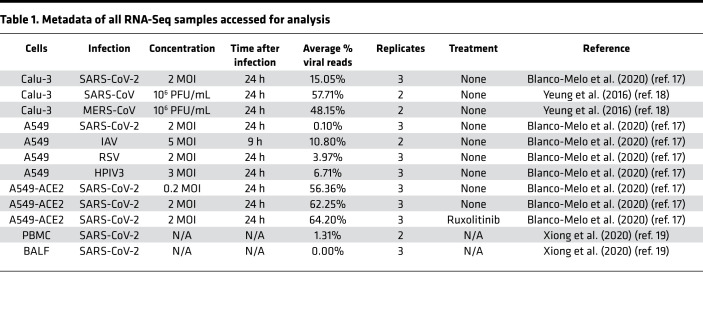
Metadata of all RNA-Seq samples accessed for analysis

## Version 1 11/03/2021

In-Press Preview

## Version 2 12/22/2021

Electronic publication
