# Supplementary material for: SARS-CoV-2 infection mediates differential expression of human endogenous retroviruses and long interspersed nuclear elements
Source: JCI Insight. 2021 Dec 22;6(24):e147170. doi: 10.1172/jci.insight.147170 (PMC8783694; doi:10.1172/jci.insight.147170)
Supplement: Supplemental data [file jciinsight-6-147170-s066.pdf]

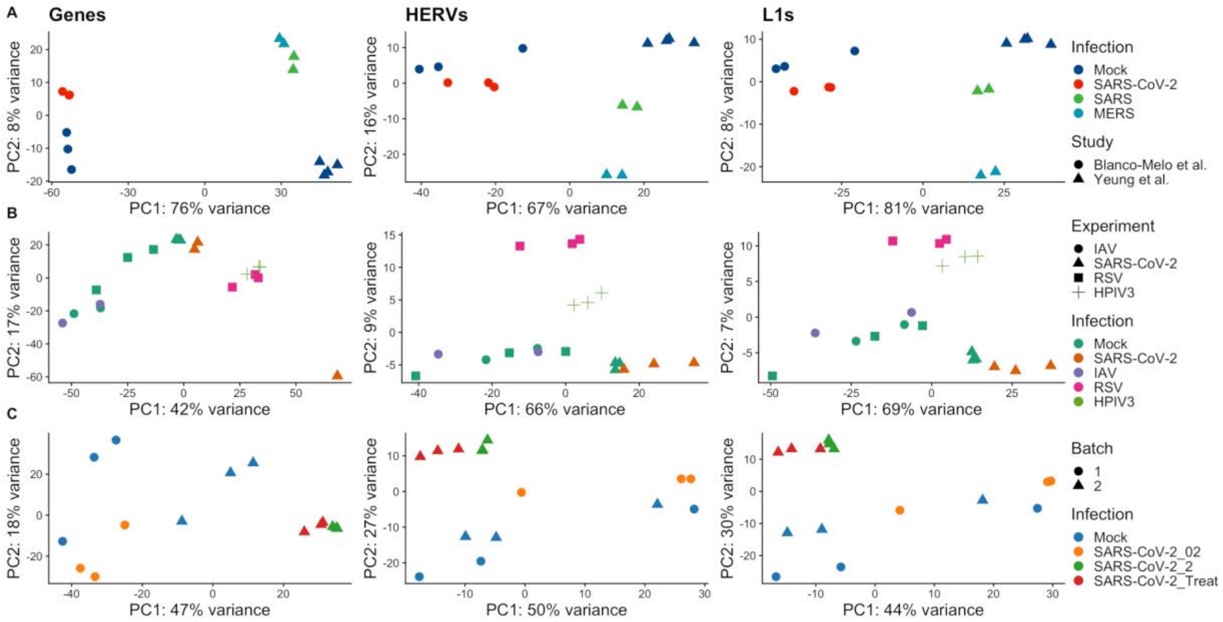

Supplemental Figure 1.
PCA plots of differences in transcriptome, retrotranscriptome and LINE-1 expression across various *in vitro* models of viral infection. (A) Clustering of samples based on expression patterns in Calu-3 cells and (B) in A549 cells and (C) A549-ACE2 cells.

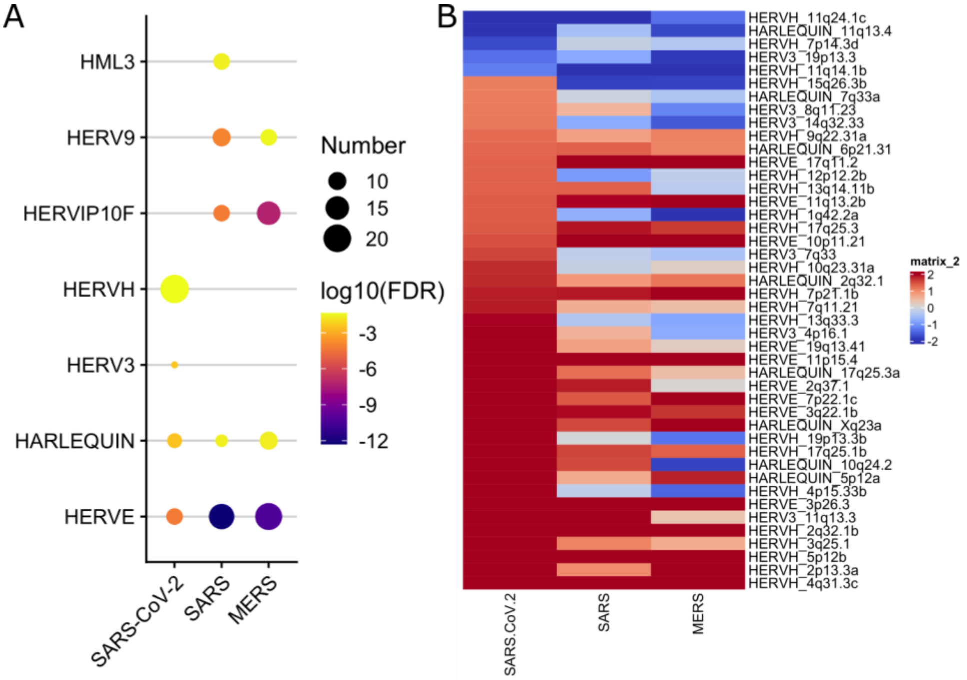

Supplemental Figure 2.
Enriched DE HERV families induced by infection of Calu-3 cells with related coronaviruses (SARS-CoV-2, SARS and MERS). (A) Dotplot expression profiles of HERV families in different infection models with plot size illustrating number of HERV elements. (B) Heatmap of differentially expressed HERV loci from HERV families enriched in SARS-CoV-2 infection colored for degree of downregulation (blue) or upregulation (red) in comparison to mock expression.

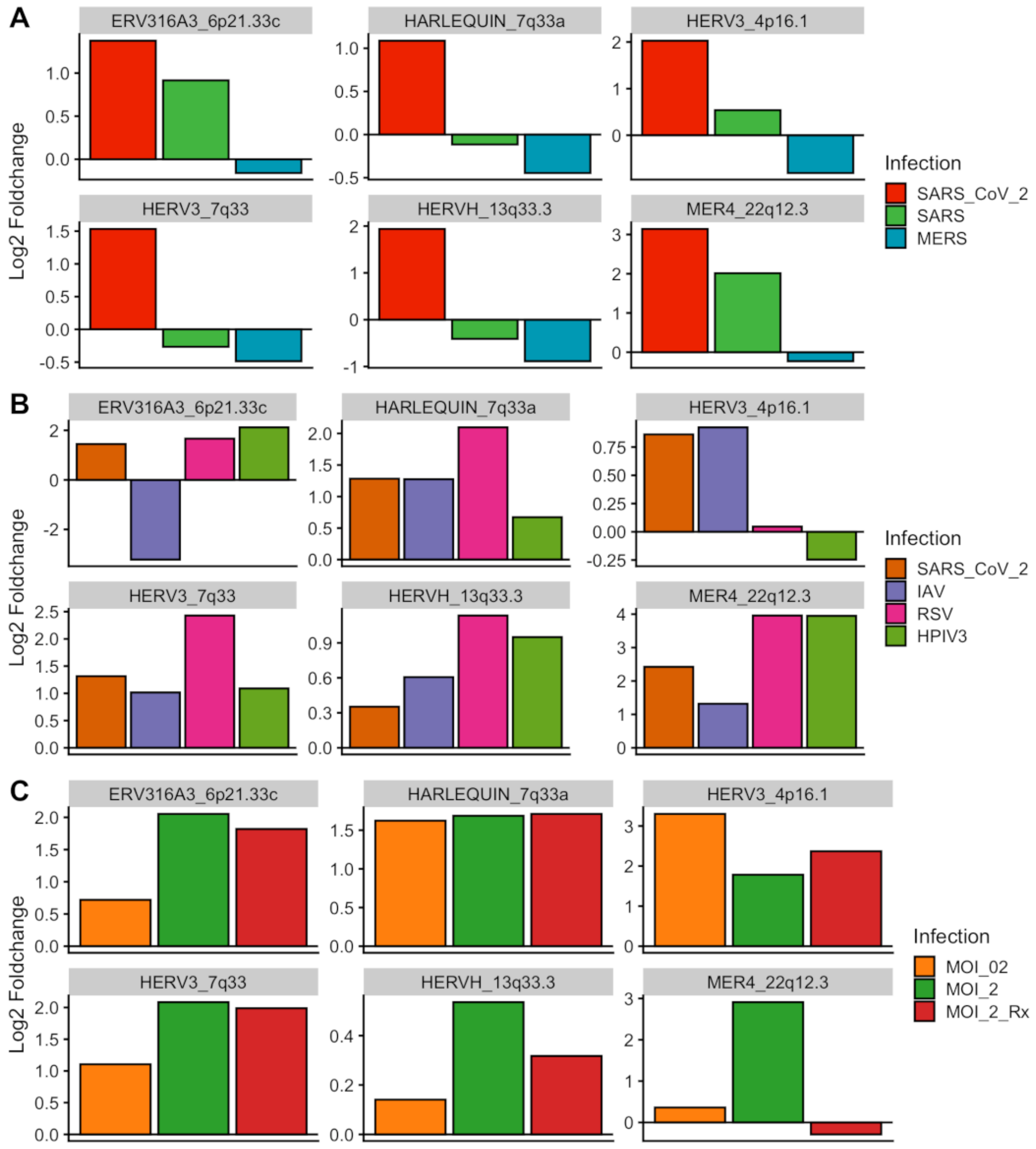

Supplemental Figure 3.
Bar plots of log2fold expression changes of selected HERVs between virus infected and mock samples from (A) Calu-3 cells, (B) A549 cells and (C) A549-ACE2 cells.

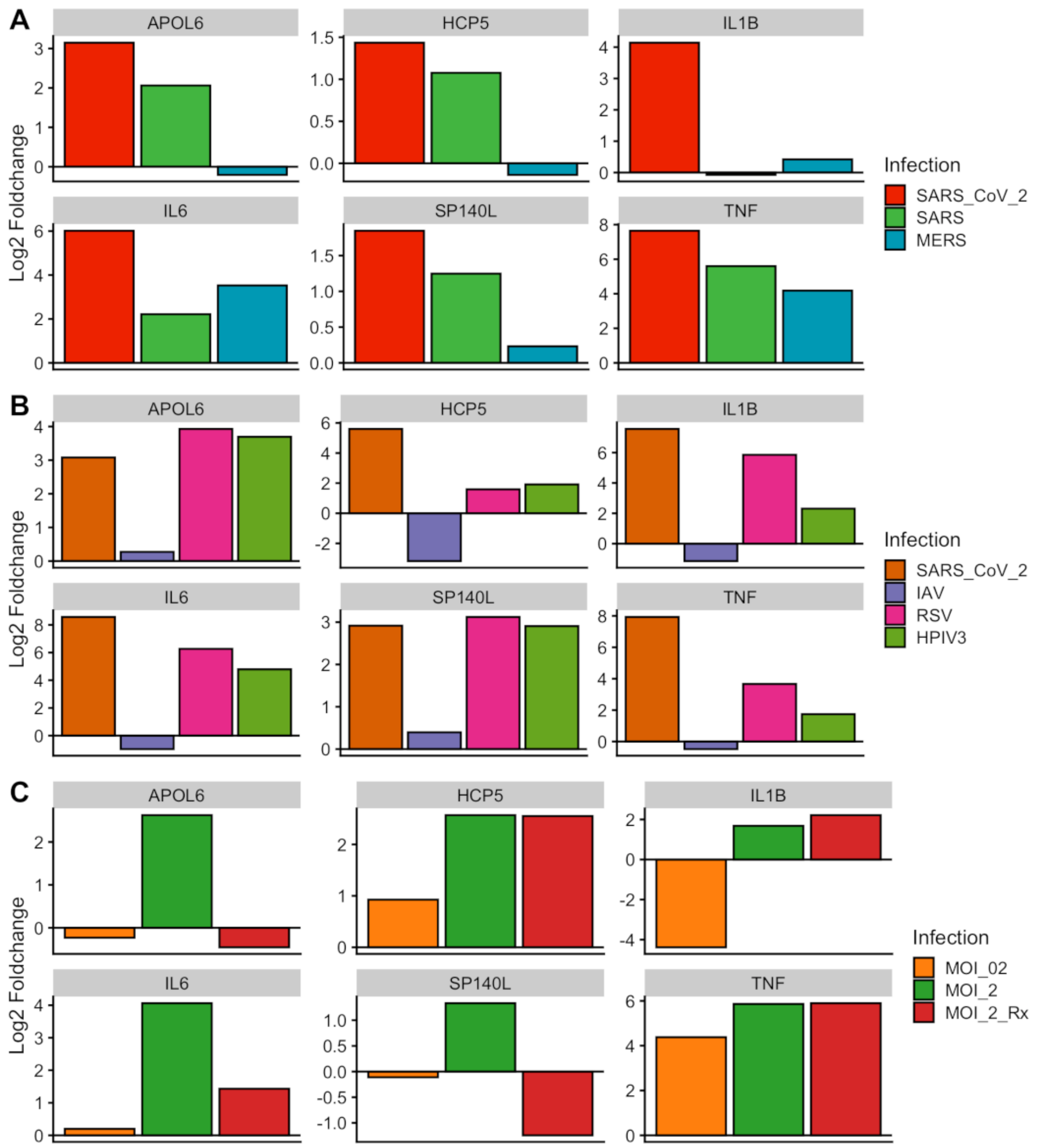

Supplemental Figure 4.
Bar plots of log2fold expression changes of selected annotated genes between virus infected and mock samples from (A) Calu-3 cells, (B) A549 cells and (C) A549-ACE2 cells.

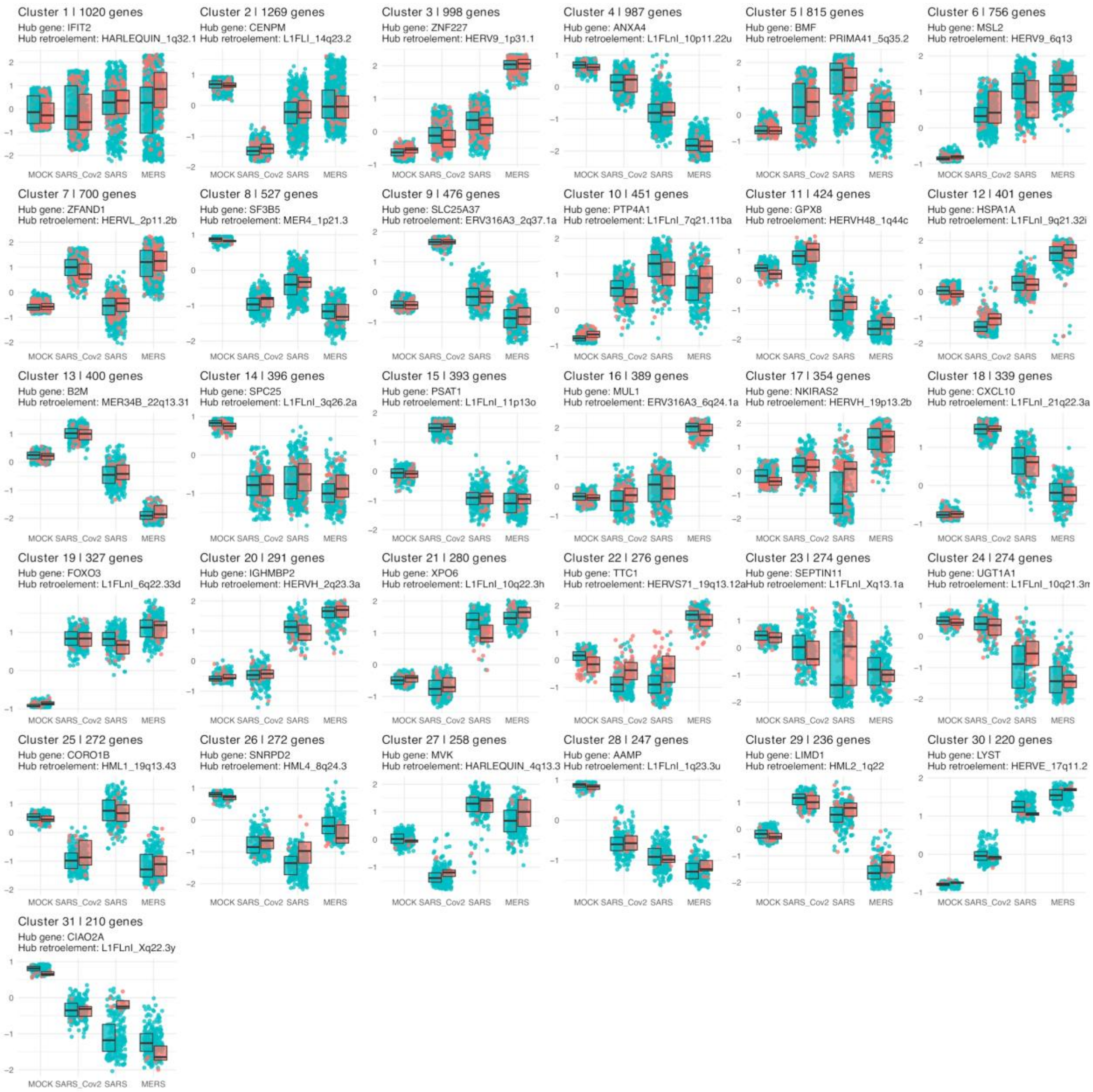

Supplemental Figure 5.
Expression trajectories for each of the 31 gene clusters produced from Calu3 lung adenocarcinoma cell transcriptomic data in four different infection conditions. For each cluster, the scaled mean expression value (z-score) for each gene in each group is plotted as a point. Box plots show the median expression value (dark line) and the first and third quartiles (edges) of the cluster's gene expression values in each infection group.

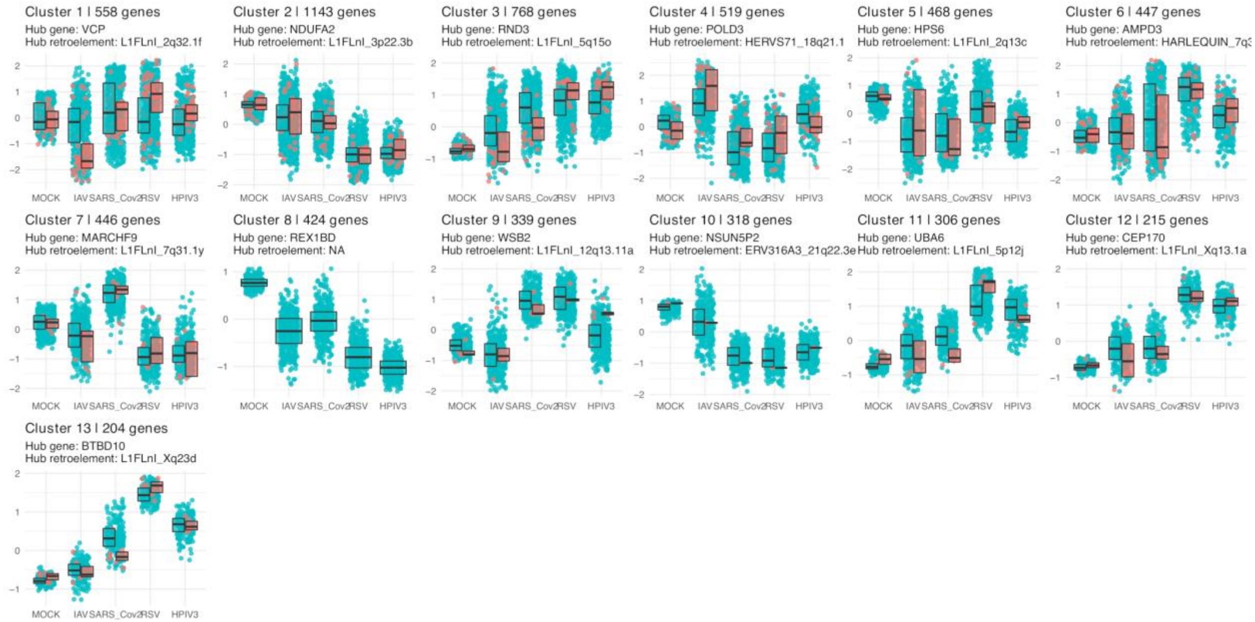

Supplemental Figure 6.

Expression trajectories for each of the 13 gene clusters produced from A549 lung adenocarcinoma cell transcriptomic data in five different infection conditions. For each cluster, the scaled mean expression value (z-score) for each gene in each group is plotted as a point. Box plots show the median expression value (dark line) and the first and third quartiles (edges) of the cluster's gene expression values in each infection group.

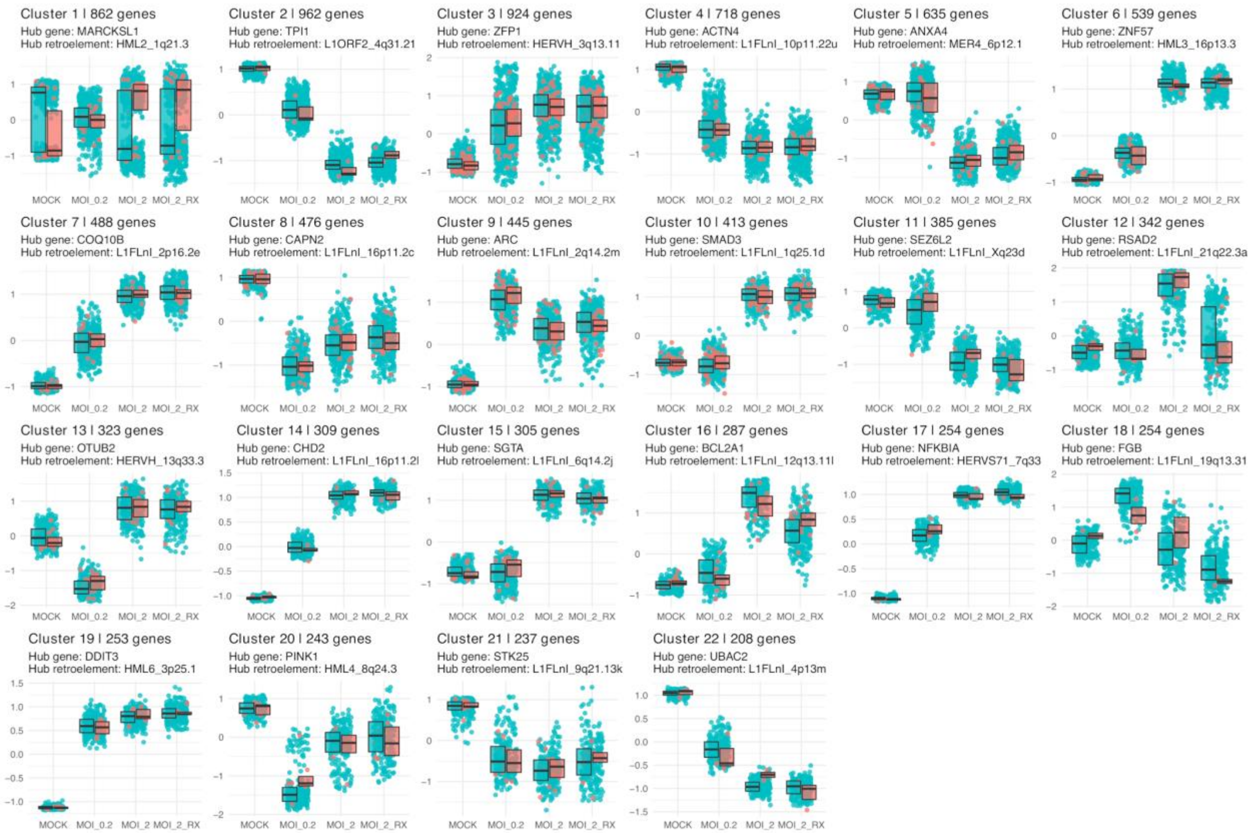

Supplemental Figure 7.

Expression trajectories for each of the 22 gene clusters produced from A549 lung adenocarcinoma cells transduced with a vector expressing human ACE2 receptor (A549-ACE2) transcriptomic data in five different infection conditions. For each cluster, the scaled mean expression value (z-score) for each gene in each group is plotted as a point. Box plots show the median expression value (dark line) and the first and third quartiles (edges) of the cluster's gene expression values in each infection group.

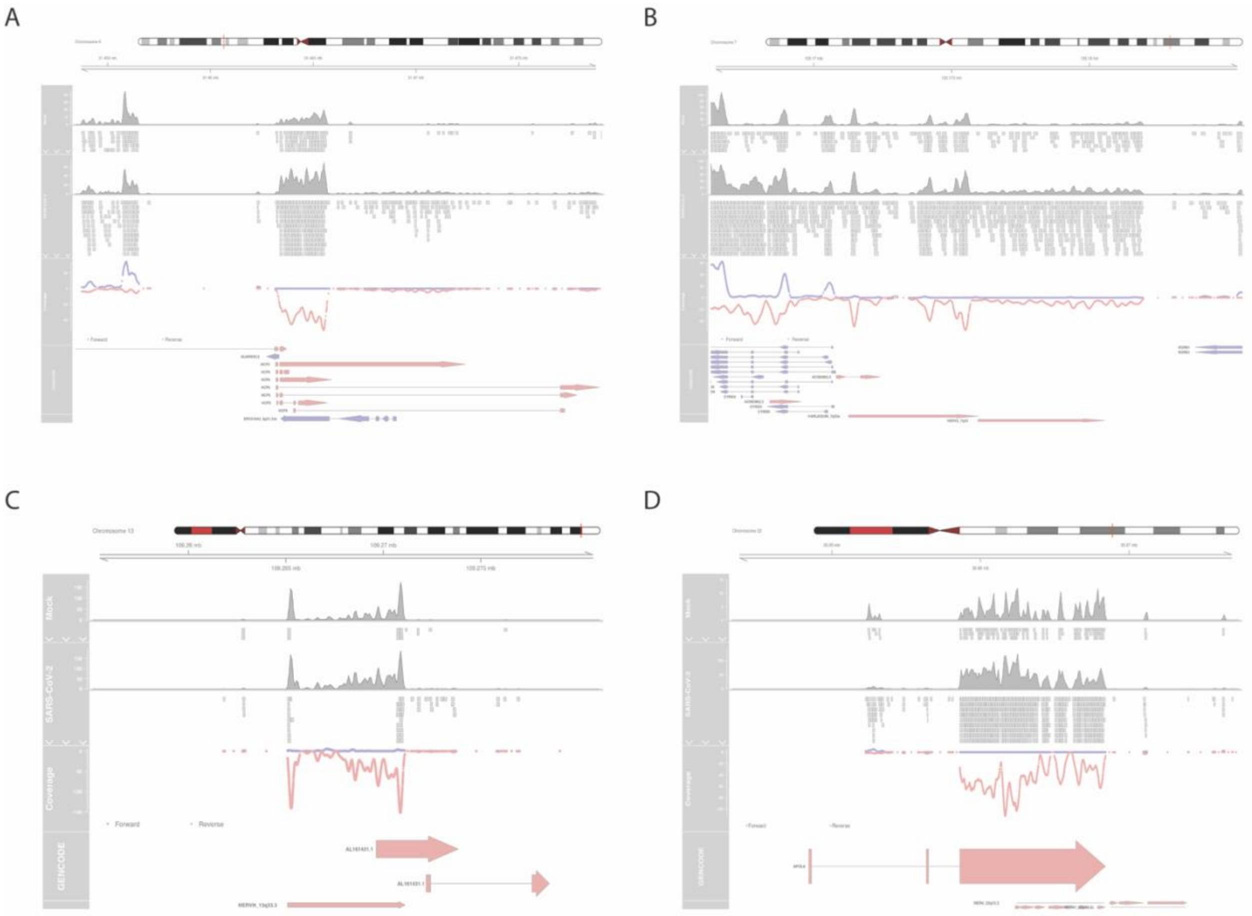

Supplemental Figure 8.
Genome viewer visualizations of selected HERVs between mock and virus infected samples showing chromosomal location, transcript coverage, strandedness of transcripts, and gene-retroelement sequence overlap for (A) HCP5 and ERV316A3 at locus 6p21, (B) CYREN, HARLEQUIN and HERV3 at locus 7q33 (C) HERVH at locus 13q33 and (D) APOL6 and MER4 at locus 22q13.

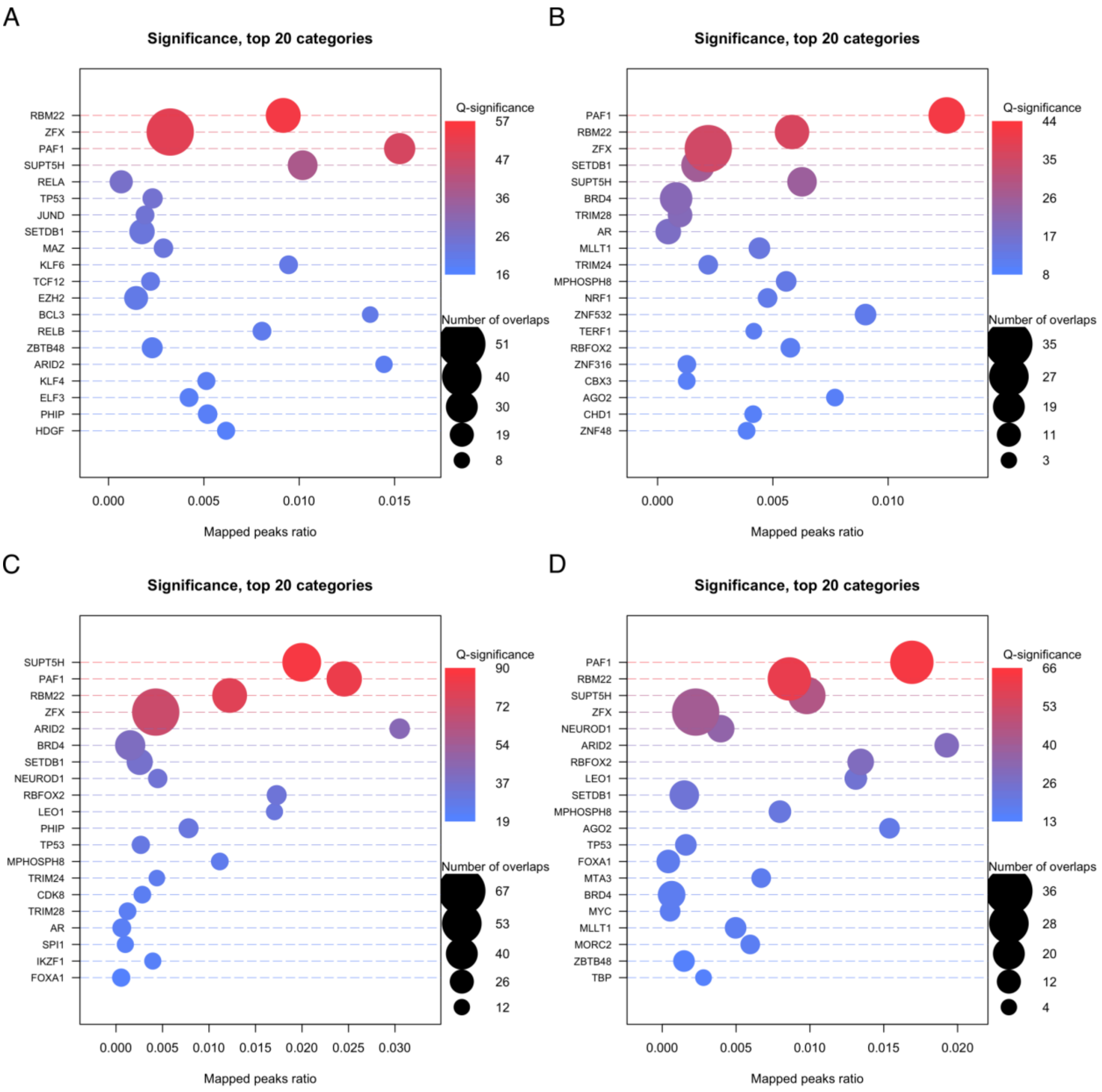

Supplemental Figure 9.
Dot plots of enrichment of binding sites for transcription factors derived from the ReMap2020 ChIP-seq database and R package for differentially expressed retroelements versus all retroelements within the genome for SARS-CoV-2 infection of (A) Calu-3, (B) A549, (C) A549-ACE2 at MOI 2 and (D) A549-ACE2 at MOI 0.2.

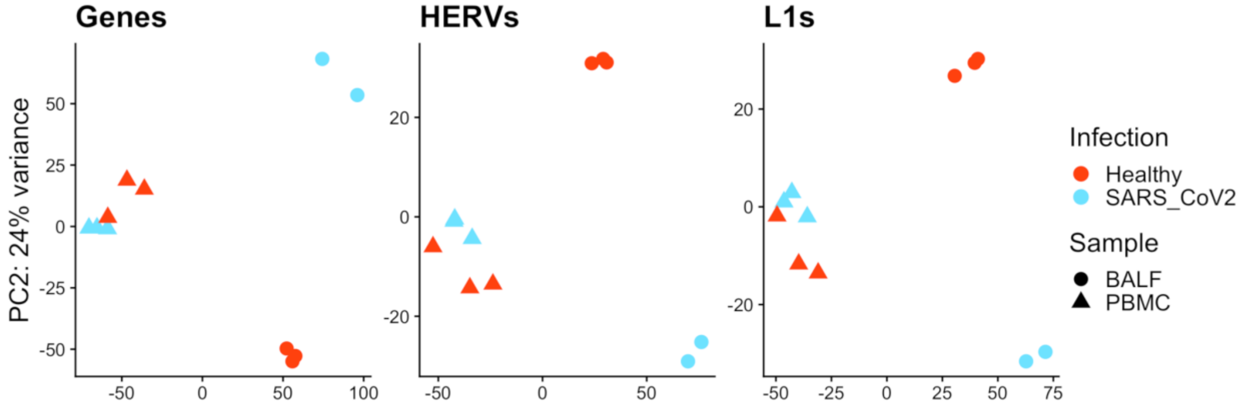

Supplemental Figure 10.
PCA plots of differences in transcriptome, retrotranscriptome and LINE-1 expression between uninfected healthy samples and samples from COVID-19 patients.
